# Dual Functions of Polyamines in Shaping Host-Specific Pathogen Dynamics

**DOI:** 10.3390/pathogens15070695

**Published:** 2026-06-30

**Authors:** Xolani H. Makhoba

**Affiliations:** Department of Life and Consumer Sciences, College of Agriculture and Environmental Sciences, University of South Africa (UNISA), Florida Campus, Roodepoort 1709, South Africa; makhoxh@unisa.ac.za

**Keywords:** polyamines, human host, putrescine, spermidine, spermine, malaria, COVID-19

## Abstract

Polyamines such as putrescine, spermidine, and spermine play essential roles in most living organisms. They regulate fundamental processes, like cell proliferation, differentiation, growth, gene expression (DNA/RNA stability, transcription, and translation), and signal transduction. As important regulators, polyamines influence development, stress responses, and the progression of health and disease, including cancer and aging. These positively charged molecules have been extensively studied for decades. In humans, polyamines are often researched as potential therapeutic targets for diseases such as malaria and, more recently, COVID-19. Obligate parasites, such as *Plasmodium falciparum*, and severe acute respiratory syndrome coronavirus 2 (SARS-CoV-2), rely on host cellular machinery for survival, replication, and growth. Notably, both hosts and pathogens need polyamines to sustain these processes. This review summarizes current advances in understanding the roles of polyamines in humans, viruses, and obligate parasites. It also explores strategies to prevent pathogens from hijacking host polyamine metabolism as a way toward developing novel therapeutic interventions.

## 1. Introduction

**Figure 1:**
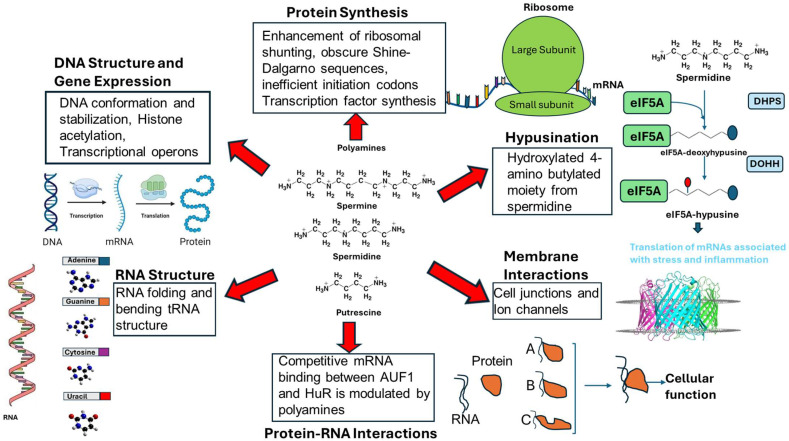
Diverse roles of polyamines in the cellular system. Polyamines such as spermine, spermidine, and putrescine, as polycationic molecules, are involved in various functions, including protein-RNA interactions, membrane interactions, hypusination, protein synthesis, DNA structure, gene expression, and RNA structure.

Polyamines are organic compounds containing two or more amino groups and are found in most living organisms. Research on polyamines dates back to 1678, when Antonie van Leeuwenhoek first observed crystalline substances in human semen, which were later identified as spermine [1,2]. Over the centuries, scientists discovered related molecules like spermidine and putrescine, gradually revealing their crucial roles in cell growth, gene regulation, and microbial survival. Due to their positive charge and low molecular weight, polyamines readily bind to DNA and RNA, influencing gene expression, protein synthesis, and replication [3,4,5,6] (Figure 1). In humans, polyamines are produced both from dietary sources and by intestinal microbiota. The biosynthetic pathway begins with amino acids L-ornithine and L-methionine, which are derived from arginine via arginase [7,8]. Putrescine is then formed from L-ornithine by the rate-limiting enzyme ornithine decarboxylase (ODC). Subsequently, spermidine and spermine are made via spermidine synthase, which transfers an aminopropyl group from S-adenosylmethionine (SAM) [9,10,11]. Beyond their roles in cellular processes, polyamines are also thought to act as molecular chaperones, enabling organisms such as plants to withstand harsh environmental conditions [12,13,14]. Plants are often exposed to environmental stressors like drought, salinity, and extreme temperatures. Polyamines are key molecules in plant adaptive responses to these abiotic challenges. Their main role is to act as molecular chaperones, stabilizing proteins and maintaining cellular structures under stress. Additionally, polyamines regulate reactive oxygen species (ROS), reducing oxidative damage and increasing the plant’s resilience to environmental pressures. The importance of polyamines, however, extends well beyond plants. In humans, they are essential for cell growth, DNA and RNA stability, protein synthesis, and the regulation of oxidative stress. At the same time, dysregulated polyamine metabolism has been linked to aging, cancer, and infectious diseases, making them both vital for health and potential targets for therapy. Pathogens also exploit polyamines for their survival. In Plasmodium, the parasite responsible for malaria, polyamines are essential for rapid replication within host cells. Similarly, viruses such as SARS-CoV-2 depend on host polyamine metabolism to stabilize viral RNA and boost replication efficiency. Limiting polyamine availability has been shown to hinder pathogen development, highlighting their role as a vulnerability in infectious disease biology. Therefore, this review will focus on two specific human pathogens, Plasmodium and SARS-CoV-2, to explore the crucial role of polyamines. By examining their functions in humans and pathogens, we can better understand how these small molecules serve as both protectors of life and enablers of disease.

There are five species of Plasmodium known to infect humans: *P. vivax*, *P. knowlesi*, *P. malariae*, *P. ovale*, and *P. falciparum*. Among these, *P. falciparum* is the most virulent, responsible for severe malaria and the majority of malaria-related deaths worldwide. *P. vivax* is widespread in Asia and Latin America and is notable for its ability to form dormant liver stages (hypnozoites), which can cause relapses months after the initial infection. *P. malariae* typically produces a milder but chronic form of malaria, with infections that may persist for years if untreated. *P. ovale*, found mainly in West Africa and some Pacific regions, also forms hypnozoites and is associated with relapsing infections. Finally, *P. knowlesi*, originally a parasite of macaques, has emerged as a significant zoonotic threat in Southeast Asia, particularly Malaysia, where it can cause rapidly progressing malaria in humans. *Plasmodium falciparum*, the causative agent of malaria, remains a major global health threat, particularly in developing countries [15]. In 2023, an estimated 249 million cases and 608,000 deaths were reported worldwide, with Sub-Saharan Africa accounting for 94% of the burden. Alarmingly, around 80% of malaria-related deaths occur among children under five years of age and pregnant women. The development of alternative and effective antimalarial drugs is highly challenging due to increasing resistance against current treatments. Consequently, malaria urgently requires innovative therapeutic strategies, especially given the vulnerability of young children and pregnant women [16]. Polyamines represent a promising drug target due to their unique bifunctional enzyme structures in the malaria parasite. In humans, polyamine biosynthesis is carried out by separate enzymes: ornithine decarboxylase (ODC) and S-adenosylmethionine decarboxylase (AdoMetDC) [17,18]. In contrast, *Plasmodium falciparum* possesses a bifunctional enzyme in which ODC and AdoMetDC are fused. These enzymes are indispensable for parasite proliferation, replication, and growth, making them attractive candidates for novel antimalarial drug development. Furthermore, polyamines may play an undefined chaperone role in *P. falciparum*, assisting parasite proteins in proper folding during replication within infected red blood cells. This potential function, critical for parasite survival, warrants further investigation as a pathway for alternative therapeutic strategies against malaria [19,20,21].

Over the past two decades, several viral outbreaks have highlighted the global threat of infectious diseases. Severe acute respiratory syndrome coronavirus-1 (SARS-CoV-1) emerged in 2002, followed by Middle East respiratory syndrome coronavirus (MERS-CoV) in 2012 [22,23]. More recently, severe acute respiratory syndrome coronavirus-2 (SARS-CoV-2), the virus behind coronavirus disease 19 (COVID-19), has become the most widespread, resulting in the highest number of reported cases and deaths to date [24,25]. To combat COVID-19, novel vaccines and drug repurposing strategies were rapidly developed, and several vaccines were approved for human use. However, viral evolution, including the emergence of variants such as Omicron and Lambda, continues to pose challenges, underscoring the need for innovative treatments and preparedness for future outbreaks [26].

Unlike *P. falciparum*, SARS-CoV-2 cannot synthesize polyamines independently. Instead, it hijacks (manipulates) host-derived polyamines to support its replication and growth, highlighting the critical role of polyamine metabolism in pathogen survival and the potential to target this pathway for therapeutic intervention. Although malaria parasites, coronaviruses, and immune cells represent distinct biological systems, they converge on a common reliance on host polyamine metabolism. This review integrates these perspectives to propose polyamine pathways as a unifying axis of host–pathogen interaction.

### 1.1. Sources and Biosynthesis of Polyamines in Humans

**Figure 2:**
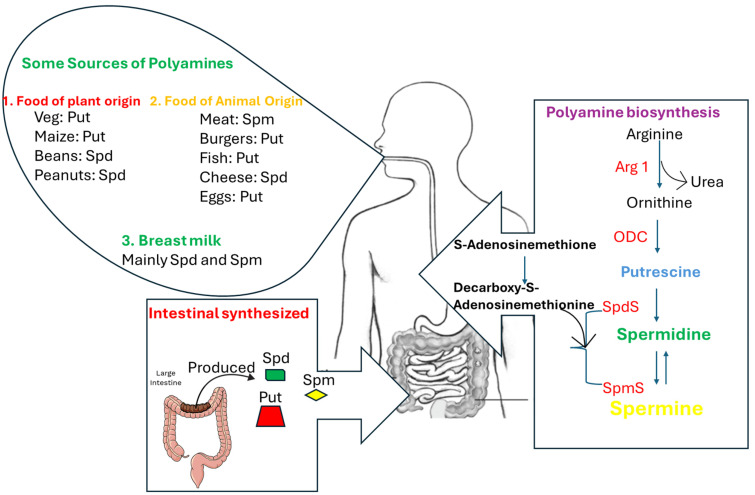
Polyamine sources and their biosynthesis in humans. Polyamines in humans come from both dietary sources and internal biosynthesis. The body synthesizes them mainly from amino acids such as ornithine, while foods like meat, fish, vegetables, and certain fermented products also contribute to polyamine intake. Abbreviations and their definitions: Put (putrescine), Spd (spermidine), Arg1 (Arginine 1), ODC (ornithine decarboxylase), Spds (spermidine synthase), and SpmS (spermine synthase).

Foods of both animal and plant origin are key sources of polyamines, which can exist in free or conjugated forms. In plant-based foods, phenolic compounds represent a type of conjugated polyamines. Major polyamines such as spermidine and spermine are naturally present in raw plant and animal tissues, while putrescine is often produced through the activity of fermentative or contaminating microorganisms [27,28,29] (Figure 2). Spermidine and spermine may also come from bacteria, especially in fermented products, indicating that processing and storage conditions can significantly influence polyamine levels. Dietary polyamines play various roles in human health and are vital for preventing complex diseases like heart disease and diabetes [30,31]. A study by Muñoz-Esparza and colleagues in 2021 examined the presence of polyamines in different food sources. For example, wheat germ was found to contain the highest levels of polyamines, at 440.6 mg/kg. In plant-based foods, concentrations around 90 mg/kg were observed in mushrooms, green peppers, peas, citrus fruits, broad beans, and tempeh, with spermidine showing the highest content, ranging from 54 to 109 mg/kg [31]. In animal-derived foods, putrescine was detected at levels between 42 and 130 mg/kg, particularly in milk, blue and hard cheeses, and dry-fermented sausages [31].

The biosynthesis of polyamines in humans begins with the breakdown of arginine, which serves as a precursor for the formation of putrescine, spermidine, and spermine [32]. Arginine is first converted into ornithine by the enzyme arginase; ornithine is subsequently converted into putrescine by ornithine decarboxylase. Putrescine is then converted into spermidine-by-spermidine synthase, and finally, spermidine is converted into spermine through the action of spermine synthase [33] (Figure 2). In addition to endogenous synthesis, significant amounts of polyamines, namely spermidine, putrescine, and spermine, are produced in the large intestine (colon). As mentioned above, polyamines can also be ingested through the diet or synthesized internally by human cells, with the gut microbiota acting as a key source, particularly in the lower gastrointestinal tract. The gut microbiota, including genera such as Bacteroides, Fusobacterium, Enterococcus, and Lactobacillus, produces polyamines in the colon by metabolizing dietary proteins and amino acids (especially arginine). These bacterial-derived polyamines are essential for maintaining the intestinal barrier, promoting epithelial cell renewal, and regulating immune responses (e.g., strengthening barrier function and increasing anti-inflammatory macrophages) [34,35].

### 1.2. General Role of Polyamines

#### 1.2.1. Cellular Activity and Metabolism

Putrescine, spermidine, and spermine are the primary polyamines found in mammals, plants, and prokaryotes. Their positively charged structures allow them to interact with biomolecules such as RNA and DNA, stabilizing these molecules, which helps promote replication and regulates RNA transcription. There are clear links between cellular activity and polyamine metabolism, as these processes convert nutrients into energy and essential building blocks vital for life, growth, repair, and homeostasis. A range of enzyme-catalyzed reactions occur in both anabolic and catabolic pathways [36,37].

#### 1.2.2. Health and Aging

Polyamines play a crucial role in maintaining cellular health; however, their levels decrease with age, a change associated with the onset of various complex diseases. Consequently, several studies have explored polyamine supplementation as a strategy to extend lifespan in animal models, showing increased autophagy and improved cellular defense mechanisms. Evidence suggests that dietary intake of polyamines, particularly spermidine, can help reduce age-related impairments such as weakened immunity and memory loss. These findings emphasize the potential of polyamines as anti-aging nutrients, although further human studies are needed to validate their benefits and establish optimal intake levels [38,39].

#### 1.2.3. Immune System Modulation

The balance between inflammation, autoimmunity, and immunosuppression is essential for proper immune system function, with polyamines acting as molecular switches in this regulation. They play a key role in the activation and proliferation of B and T lymphocytes. Moreover, polyamines are necessary for the post-translational modification of eukaryotic initiation factor 5A (eIF5A) through hypusination, a process vital for T-cell activity and autophagy. Macrophage polarization is largely driven by spermidine, which promotes an M2-like (anti-inflammatory/reparative) phenotype that supports tumor growth. Conversely, reduced polyamine levels can shift macrophages toward an M1-like (pro-inflammatory) phenotype. In autoimmune diseases such as Systemic Lupus Erythematosus (SLE), systemic polyamine levels are often diminished, which correlates with heightened inflammatory responses. However, in some cases, such as Th17-mediated autoimmunity, polyamines can exacerbate disease, and their inhibition may provide protective effects [40,41].

#### 1.2.4. Chemical Chaperone Activities of Polyamines

Polyamines (Putrescine, Spermidine, and Spermine) act as chemical chaperones that protect proteins and nucleic acids from denaturation and aggregation, especially under environmental stresses such as heat, salt, and drought. Their chaperone-like activity (CLA) stabilizes protein structures and prevents misfolding under stress. Polyamines reduce thermal and oxidative aggregation of proteins, notably by decreasing turbidity caused by denatured proteins such as malate dehydrogenase (MDH). They bind to anionic macromolecules (DNA, RNA, proteins, phospholipids) to maintain their structural integrity. In plants, they help protect the photosynthetic machinery (e.g., Rubisco) from degradation. Polyamines work synergistically with Heat Shock Proteins (HSPs) and other chaperones to assist in protein folding. They are known to influence the activity of Hsp90. Additionally, they enhance tolerance to salt, drought, and high temperatures by regulating ROS scavenging, reducing chlorophyll degradation, and stabilizing membranes. They also act as acid tolerance factors, maintaining cellular pH by serving as precursors for proton consumption. Spermine and spermidine are essential for the activity of eukaryotic translation initiation factor 5A (eIF5A), facilitating proper protein synthesis [12,42,43].

## 2. Polyamines in Parasite Infections

### 2.1. Polyamine Biosynthesis in Plasmodium falciparum

**Figure 3:**
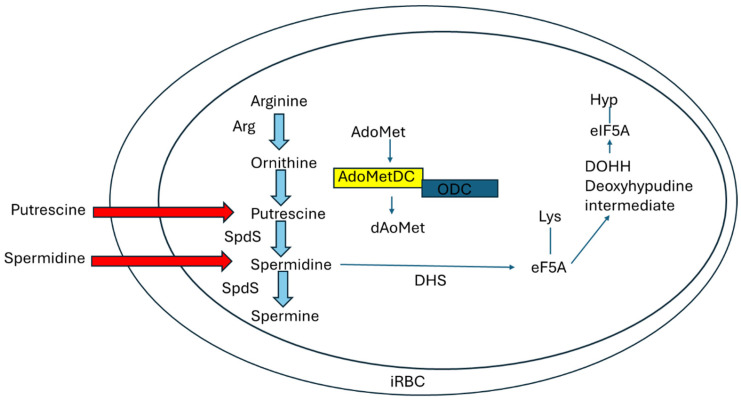
Biosynthesis of polyamines in *P. falciparum*. *Plasmodium* possesses a core polyamine (PA) biosynthetic pathway that includes ornithine decarboxylase (ODC), S-adenosylmethionine decarboxylase (AdoMetDC), and spermidine synthase (SpdS). Distinctive features of this pathway in *Plasmodium* are the presence of a bifunctional AdoMetDC and a spermidine synthase capable of producing spermine (Spm). In addition, the parasite can utilize putrescine (Put) and spermidine (Spd) derived from the host red blood cell (RBC) salvage pathway. Hypusine biosynthesis is highly conserved across species. In this process, deoxyhypusine synthase (DHS) catalyzes the transfer of an aminopropyl moiety to lysine-50 of the precursor protein, while deoxyhypusine hydroxylase (DOHH) introduces a hydroxyl group at carbon-9 of the modified side chain. Abbreviations and definitions: Arg (arginine), AdoMet (S-adenosylmethionine), Hip (hypusine), eIF5A (eukaryotic Initiation Factor), and Lys (Lysine).

Malaria remains a leading cause of mortality in Sub-Saharan Africa, with an estimated 282 million cases and 6100 deaths reported globally [44]. Pregnant women and children under five are most vulnerable due to their weaker immune systems. The rising prevalence of drug-resistant malaria strains worldwide underscores the urgent need for novel therapeutic strategies. *Plasmodium falciparum* is the main cause of malaria among the five *Plasmodium* species [45,46]. The first step in drug development is identifying suitable molecular targets, which can be done through computational approaches. The bifunctional polyamine biosynthetic pathway in *P. falciparum* is an attractive target because it differs significantly from the human pathway [47,48]. Notably, two enzymes are encoded within a single open reading frame, a unique feature distinguishing the parasite from its human host. This structural arrangement enables a unified targeting approach, unlike in humans, where the enzymes are separate. Polyamine biosynthesis in *P. falciparum* involves the decarboxylation of ornithine-by-ornithine decarboxylase (ODC) and S-adenosylmethionine decarboxylase (AdoMetDC), leading to the formation of putrescine, spermidine, and spermine (Figure 3). Additionally, spermidine synthase catalyzes the synthesis of both putrescine and spermidine. During the asexual intraerythrocytic stage of *P. falciparum*, intracellular polyamine levels are high, with spermidine reaching about 6 mM in mature trophozoites and putrescine around 0.5 mM [49]. Given this dependency, drugs like difluoromethylornithine (DFMO) have been proposed as potential candidates for malaria treatment, specifically targeting polyamine biosynthesis.

Polyamine analogues are designed to disrupt metabolism, interfere with RNA and DNA functions, and inhibit polyamine transport systems. At least 22 compounds from various classes of polyamine analogues have been tested for their ability to inhibit *Plasmodium falciparum* replication and growth both *in vitro* and *in vivo* [50]. Studies have reported a 10- to 20-fold increase in polyamine levels during the parasite’s transition from the ring stage to schizonts in infected red blood cells, highlighting the parasite’s dependence on polyamines for development. Inhibitors of this pathway have been shown to lower polyamine levels, which consequently cause transcriptional arrest [51]. Therefore, polyamines are promising drug targets for further exploration and small-molecule development, given their essential role in *P. falciparum* survival.

### 2.2. Hypusination of eIF5A and Its Role

Hypusination is a rare, highly specific post-translational modification in which spermidine donates an aminobutyl group to a lysine residue in the eukaryotic initiation factor 5A (eIF5A). This process, catalyzed by deoxyhypusine synthase (DHS) and deoxyhypusine hydroxylase (DOHH), is essential for converting eIF5A into its active form [52]. Hypusinated eIF5A plays a critical role in translation elongation, particularly in resolving ribosomal stalling at polyproline sequences, thereby ensuring efficient protein synthesis. Beyond translation, hypusination of eIF5A influences cell proliferation, stress responses, mitochondrial function, and immune regulation. Dysregulation of this pathway has been implicated in cancer progression, viral replication, metabolic disorders, and neurodegenerative diseases such as ALS [53]. Therapeutically, inhibitors of hypusination (e.g., GC7) are being explored as anticancer, antiviral, and cytoprotective agents, though challenges remain because polyamines are essential to normal physiology. Overall, hypusination represents a pivotal molecular mechanism linking polyamine metabolism to health and disease [54].

### 2.3. Polyamine Transport System

Polyamine transport systems are essential for maintaining intracellular levels of putrescine, spermidine, and spermine, small polycations required for cell growth, proliferation, and stress adaptation. Because polyamines cannot freely diffuse across membranes, cells rely on specialized transporters, including solute carrier (SLC) proteins and endocytic pathways, to regulate uptake and export [55]. These systems are tightly controlled by cellular demand and extracellular availability, ensuring homeostasis under normal conditions. Importantly, pathogens such as viruses and parasites exploit polyamine transport to enhance replication, highlighting its role as a critical interface between host metabolism and infection. Inhibiting polyamine transport has been shown to reduce pathogen survival, but therapeutic strategies must balance efficacy with the risk of disrupting normal immune and epithelial cell function [56,57]. Overall, polyamine transport systems represent a promising yet complex target for drug discovery, requiring deeper mechanistic insights into their regulation during health and disease.

### 2.4. The Uptake of Polyamines by P. falciparum from Human Host Cells

**Figure 4:**
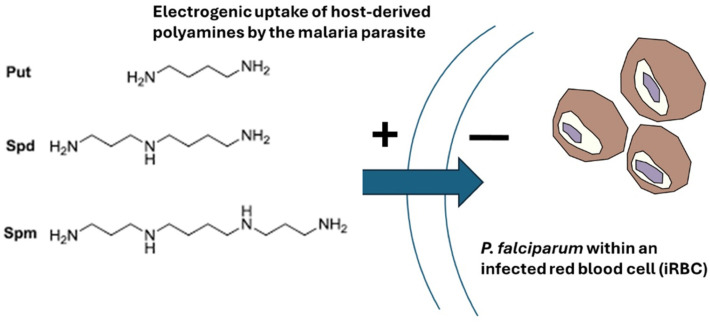
The uptake of polyamines within host-infected red blood cells is a crucial process in malaria infection. In the human host, polyamines such as putrescine (Put), spermidine (Spd), and spermine (Spm) are transported into the parasite via specific transporters. The environment inside infected red blood cells is highly negatively charged, while the outside is more positively charged. Because polyamines carry positive charges, they can readily cross into the parasite system, supporting its growth and survival.

The malaria parasite maintains high levels of polyamines, particularly spermidine and spermine, during its rapid replication within human erythrocytes. To achieve this, the parasite relies on both de novo polyamine biosynthesis and uptake from the surrounding environment, such as the host cytoplasm [58,59]. The uptake of polyamines is influenced by extracellular pH and the permeability of the parasite membrane (Figure 4). Infected red blood cells acquire a more negative charge compared to the host cell environment. As a result, positively charged polyamines are attracted to the negatively charged infected erythrocytes. Because of this, the system is considered an ideal drug target, as it involves both the synthesis and transport of polyamines.

## 3. Polyamines in Viral Infections

### 3.1. Global Emergence and Impact of Coronaviruses (2002–2019)

In 2002, the severe acute respiratory syndrome coronavirus 1 (SARS-CoV-1) was first identified after emerging from bats in China, eventually spreading among human populations [60]. A decade later, in 2012, another virus called Middle East respiratory syndrome coronavirus (MERS-CoV) appeared and infected humans [61]. In 2019, the outbreak of severe acute respiratory syndrome coronavirus 2 (SARS-CoV-2) led to coronavirus disease 2019 (COVID-19), which spread worldwide, disrupted economies, and caused millions of households to lose their income [62,63]. More than 704,753,890 COVID-19 cases were reported, with 7 million deaths globally. An urgent need arose to develop vaccines or drugs to prevent further spread of the disease worldwide [64]. Consequently, more resources were allocated toward vaccine development. Underdeveloped countries were hit hardest due to inadequate infrastructure, such as clinics and hospitals, along with a shortage of medical personnel capable of managing such a pandemic (Table 1).

### 3.2. Genomic Organization and Protein Composition of SARS-CoV-1, MERS-CoV, and SARS-CoV-2

**Figure 5:**
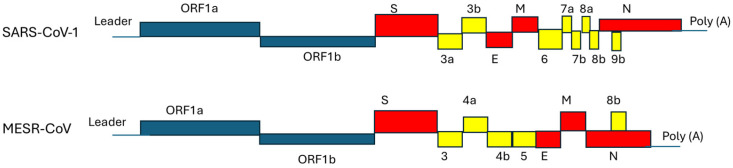
Genome structures of SARS-CoV and MERS-CoV. The single-stranded positive-sense coronavirus genomes encode the structural proteins (red), membrane (M), spike (S), envelope (E), and nucleocapsid (N), two replicase polyproteins (green), ORF1a and ORF1b, and unique accessory proteins (yellow) that perform important functions in coronavirus replication and pathogenesis, such as blocking the innate immune signaling pathway.

SARS-CoV-1 is a single-stranded, positive-sense RNA virus with a genome size of approximately 29.7 kb. Its genome contains two large open reading frames (ORF1a and ORF1b), which encode non-structural proteins (nsps 1–16). The structural proteins include the spike (S), envelope (E), membrane (M), and nucleocapsid (N). Accessory proteins are encoded by ORFs 3a, 6, 7a, 7b, 8a, 8b, and 9b. The replication strategy of SARS-CoV-1 involves ribosomal frameshifting to produce polyproteins pp1a and pp1ab, which are subsequently cleaved into individual non-structural proteins that assemble into the replication–transcription complex (Figure 5) [65]. MERS-CoV, on the other hand, has a slightly larger genome of about 30.1 kb. Like SARS-CoV-1, it is a positive-sense RNA virus with ORF1a and ORF1b encoding nsps 1–16. Its structural proteins also consist of S, E, M, and N. However, MERS-CoV possesses a distinct set of accessory proteins, including ORFs 3, 4a, 4b, 5, and 8b. Notably, accessory proteins 4a and 4b play a critical role in antagonizing host immune responses, particularly by interfering with interferon signaling (Figure 5) [60].

Generally, some viruses have genomes made of ribonucleic acid (RNA) instead of DNA. For instance, viruses such as influenza, HIV, and SARS-CoV-2 rely on RNA for replication, protein synthesis, and adaptation. SARS-CoV-2 possesses a single-stranded, positive-sense RNA genome approximately 29.9 kb, making it one of the largest RNA virus genomes [66,67]. It encodes both structural proteins, like Spike (S), Envelope (E), Membrane (M), and Nucleocapsid (N), as well as non-structural proteins. The non-structural proteins are encoded by the open reading frames 1a (ORF1a) and 1b (ORF1b) [61,68]. These regions are translated into polyproteins (pp1a and pp1ab), which are subsequently cleaved into 16 distinct non-structural proteins.

**Figure 6:**
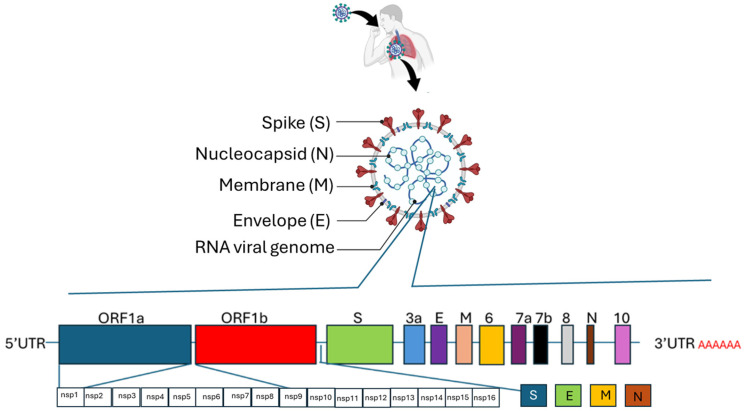
The genome structure of SARS-CoV-2 is represented as a single-stranded RNA virus. It encodes several structural proteins, including S, E, M, and N. In addition, its open reading frames (ORFs) a and b produce non-structural proteins.

The virus primarily spreads from person to person through respiratory droplets, such as those released during coughing (Figure 6). Because the virus cannot synthesize its own proteins, it hijacks the host’s cellular machinery to replicate and proliferate. Among the host components required are molecular chaperones, which assist in the folding of newly synthesized proteins [69]. Our research group has demonstrated in several publications that human molecular chaperones indeed bind to viral proteins. In addition, some studies suggest that SARS-CoV-2 may interact with erythroid progenitors, potentially contributing to dysregulated iron and hemoglobin metabolism. However, direct infection of mature erythrocytes and their role in viral spread remain unsubstantiated and require further investigation.

As a result, the virus exploits host chaperones to ensure proper protein folding, while polyamines facilitate its proliferation, differentiation, and growth. In addition, polyamines are critical for cellular viability and play a crucial role in viral attachment and replication. SARS-CoV-2 relies on host molecular chaperones such as heat shock protein 70 (HSP70) and heat shock protein 90 (HSP90) to regulate protein folding and stabilize viral proteins during replication. Importantly, this cooperation suggests that both polyamines and heat shock proteins act as cooperative partners manipulated by the virus, particularly through viral NSP13, which interacts with both polyamines and HSPs. This mechanism enables the virus to spread further by infecting additional erythrocytes [70].

**Figure 7:**
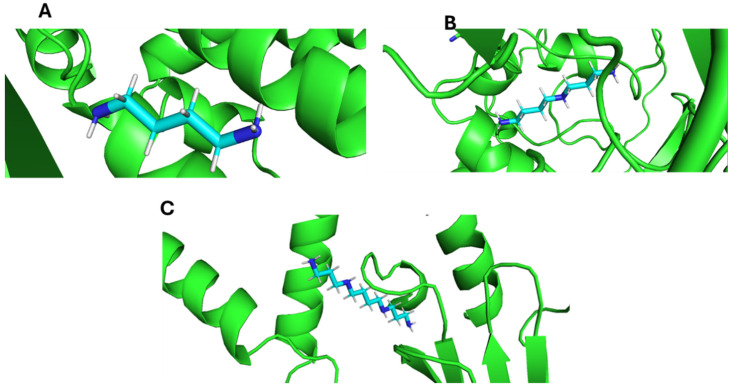
Demonstrates the molecular docking of putrescine, spermidine, and spermine with SARS-CoV-2 structural proteins. (**A**) Putrescine interacts with the Spike protein, (**B**) Spermidine interacts with the Nucleocapsid protein, and (**C**) Spermine interacts with the Membrane protein. These interactions suggest a stabilizing role for polyamines in viral protein folding, supporting the hypothesis that host polyamines are co-opted during infection.

Polyamines play a crucial role in protein synthesis due to their positive charge, and viruses such as SARS-CoV-2 exploit host polyamines to facilitate protein binding, folding, and translation [71,72]. To investigate this hypothesis, molecular docking was performed using selected viral structural proteins, spike, nucleocapsid, and membrane, whose functions are summarized in Table 2. The results, shown in Figure 7, demonstrate that putrescine interacts with the spike protein, spermidine with the nucleocapsid protein, and spermine with the membrane protein. These interactions suggest that polyamines stabilize viral protein conformations, enhance RNA binding, and promote replication. While computational evidence is compelling, experimental validation remains limited, and further biochemical studies are required to confirm these predicted interactions. Overall, the findings highlight polyamines as modulators of coronavirus protein activity and potential therapeutic targets. Ongoing biophysical studies in our laboratory aim to further characterize these interactions and develop agents that inhibit polyamine–viral protein binding. Such strategies could disrupt viral replication and pathogenicity, not only in SARS-CoV-2 but also in other viral families that depend on host polyamine pathways [73].

Drug discovery and development through computational studies play a critical role in addressing emerging and existing complex diseases. A full understanding of how SARS-CoV-2 hijacks human polyamines is essential for developing effective therapeutic strategies against COVID-19 and its long-term effects, such as post-acute COVID syndrome [74]. Moreover, these insights could enhance preparedness for future viral outbreaks. Beyond the immediate benefits, these drugs and vaccines would improve overall human health and well-being, while ensuring affordable access to treatment. This is achievable by repurposing and innovating existing drugs, thereby accelerating the creation of cost-effective therapies.

## 4. Limitations of Therapeutically Targeting Polyamine Metabolism in Human Viral Infections

Targeting polyamine metabolism as an antiviral approach faces several challenges. Since polyamines are vital for normal cell growth and maintaining balance, broad inhibition, such as with difluoromethylornithine (DFMO), can lead to toxicity in healthy tissues, especially those with rapid cell turnover. Additionally, polyamine depletion is most effective as a preventive measure because DFMO is slow-acting and needs time to reduce existing polyamine pools, making it less effective against active infections [75]. Host cells can also compensate for pathway inhibition by activating alternative routes or importing external polyamines, which reduces antiviral effectiveness. Pharmacologically, depletion effects are reversible once polyamines are replenished, and *in vivo* studies show uneven drug distribution among organs, limiting systemic success. Furthermore, because polyamine metabolism is tightly controlled, blocking a single enzyme is often insufficient and often requires combination therapies that may increase side effects. In summary, while targeting this pathway presents a promising antiviral strategy, its practical use is limited by host toxicity, delayed action, compensatory mechanisms, and distribution challenges [76]. These issues reveal the complexity of exploiting a pathway so integral to normal cellular function. Still, the reliance of various pathogens—including malaria parasites and coronaviruses—on host polyamines suggests a common weakness. By addressing these limitations and considering polyamine biology across different infections, future research may develop combination approaches or targeted treatments that hinder pathogen use of polyamines with minimal harm to the host [77].

## 5. Proposed “Cocktail” Treatment for SARS-CoV-2: Targeting the Polyamine Pathway and Molecular Chaperone Transport System

**Figure 8:**
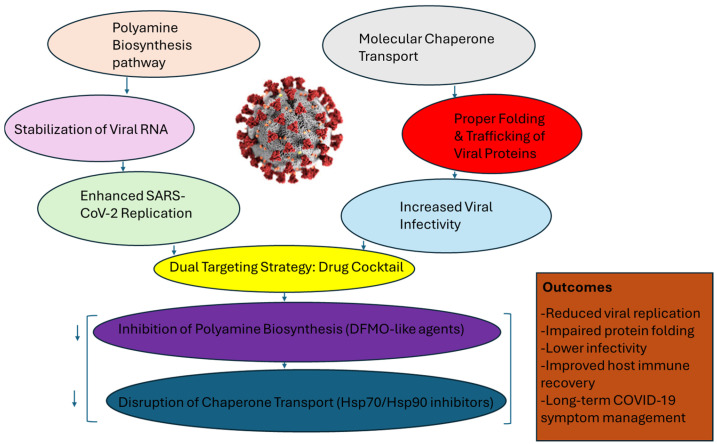
A proposed treatment for SARS-CoV-2 targeting both polyamines and molecular chaperones.

A combined therapeutic strategy that simultaneously disrupts host polyamine metabolism and interferes with molecular chaperone transport may provide a more effective approach against SARS-CoV-2. Polyamines are critical for viral RNA stabilization and protein translation, while molecular chaperones such as HSP70 and HSP90 facilitate the proper folding of viral proteins [78]. Targeting both pathways in tandem could reduce viral replication efficiency and limit pathogenicity. However, this “cocktail” approach must be carefully evaluated to balance antiviral efficacy with host safety, given the essential roles of polyamines and chaperones in normal cellular physiology. Figure 8 illustrates this dual-target strategy. On the left, polyamine metabolism is depicted: positively charged molecules such as putrescine, spermidine, and spermine interact with viral structural proteins (spike, nucleocapsid, and membrane), stabilizing their folding and enhancing replication. Inhibitors of polyamine biosynthesis or transport (e.g., DFMO, GC7) are proposed to block these interactions, thereby destabilizing viral proteins and reducing infectivity. On the right, molecular chaperones such as HSPA8 are shown assisting in the folding of the viral spike glycoprotein. Inhibiting chaperone activity with specific modulators could impair viral protein maturation and assembly.

## 6. Conclusions

Polyamines are in high demand by both human host systems and pathogens such as viruses and *Plasmodium falciparum*, underscoring their critical role in cell proliferation, differentiation, and growth. Cells infected by either viruses or *P. falciparum* exhibit markedly elevated polyamine concentrations compared to normal cells. Importantly, the fundamental differences in polyamine biosynthesis between humans and malarial parasites make these pathways attractive drug targets for malaria therapy. Viruses, which lack the ability to synthesize their own proteins and instead rely on host cellular machinery, present further opportunities to investigate how polyamines are transported from host cells to viral components. Moreover, the poorly understood relationship between polyamines and molecular chaperone proteins such as HSPs, which assist in protein folding and stabilization may be crucial for enabling both viruses and parasites to produce functional proteins. Exploring these interactions could open new avenues for therapeutic development against malaria, COVID-19, and related sequelae by disrupting the metabolic and protein folding processes that pathogens depend on for survival and replication [78].

## Figures and Tables

**Table 1:** Summary of the emergence and its impact on human hosts.

Name	Origin	Outbreak	Human impact
SARS-CoV-1	China	2002–2003	8422 cases and 916 deaths [60]
MERS-CoV	Arabian Peninsula	2012	2000 cases and 700 deaths [61]
SARS-CoV-2	Wuhan, China	2019	704,753,890 cases and 7 million deaths [62,63,64]

**Table 2:** Proteins Driving SARS-CoV-2 Development.

Names of Non-Structural Proteins	Role
Nsp1	Suppresses host immune response by inhibiting host mRNA translation and promoting mRNA degradation.
Nsp2	Modulates host cell signaling; its exact role is still under investigation.
Nsp3	Large multi-domain protein; includes papain-like protease (PLpro) for polyprotein cleavage and immune evasion.
Nsp4	Works with Nsp3 to form double-membrane vesicles for viral replication.
Nsp5	Main protease (Mpro/3CLpro); cleaves viral polyproteins into functional units.
Nsp6	Involved in autophagy regulation and formation of replication organelles.
Nsp7	Cofactor for RNA-dependent RNA polymerase (RdRp).
Nsp8	Works with Nsp7 to form the primase complex for RNA synthesis.
Nsp9	RNA-binding protein aiding replication.
Nsp10	Cofactor that stabilizes Nsp14 and Nsp16 enzymatic activity.
Nsp11	Very small peptide; function remains unclear.
Nsp12	RNA-dependent RNA polymerase (RdRp); central enzyme for viral RNA replication.
Nsp13	Helicase unwinds RNA during replication.
Nsp14	Exonuclease with proofreading activity; also has methyltransferase function.
Nsp15	Endoribonuclease (NendoU) helps evade host immune detection.
Nsp16	2′-O-methyltransferase; modifies viral RNA cap to mimic host mRNA and avoid immune recognition.
**Names of Structural Proteins**	**Role**
Membrane (M)	Shapes the viral envelope; central organizer of virus assembly.
Nucleospade (N)	Packages viral RNA genome; aids replication and transcription.
Envelope (E)	Small protein involved in virus assembly, release, and pathogenesis.
Spike (S)	Mediates binding to the host ACE2 receptor and entry into cells.

## Data Availability

No new data were created or analyzed in this study.
